# Recent Advances in the Characterization of Uropathogenic Escherichia coli Associated With Resistance and Virulence Mechanisms

**DOI:** 10.7759/cureus.108845

**Published:** 2026-05-14

**Authors:** Manasi R Mahajan, Geeta S Karande, Satish R Patil

**Affiliations:** 1 Department of Microbiology, Krishna Institute of Medical Sciences, Krishna Vishwa Vidyapeeth (Deemed to Be University), Karad, IND

**Keywords:** antimicrobial resistance, biofilm formation, carbapenem resistance, esbl, india, pcr, tertiary care hospital, urinary tract infections, uropathogenic escherichia coli, virulence genes

## Abstract

Globally, urinary tract infections (UTIs) constitute a major concern as one of the most frequent bacterial infections and a major public health concern, particularly in developing countries like India. Among the various uropathogens, uropathogenic *Escherichia coli* (UPEC) is the principal causative agent of UTIs in both community-acquired and hospital-associated settings. UPEC strains are typically identified based on the presence of specific virulence factors (e.g., fimH, pap, sfa, afa) and phylogenetic grouping (mainly groups B2 and D), along with serotyping and molecular methods such as polymerase chain reaction (PCR), reported in previous studies. The increasing emergence of antimicrobial resistance, especially among UPEC isolates, has significantly complicated the management of UTIs and contributed to higher morbidity and healthcare burden. This review summarizes published literature on UPEC, with an emphasis on antimicrobial resistance patterns, virulence gene profiles, and biofilm-forming ability described across different studies. It also highlights the application of molecular techniques, particularly PCR, as reported in previous research for the detection of key resistance genes, such as *blaTEM*, *blaSHV*, *blaCTX-M*, and *qnr*, and aminoglycoside resistance determinants, as well as virulence-associated genes including *fimH*, *pap*, and *sfa*. Several studies, including reports from India and other regions, have documented a high prevalence of multidrug-resistant UPEC strains, commonly linked to extended-spectrum beta-lactamase production and, in some cases, emerging carbapenem resistance. Several studies have reported that approximately 50-80% of UPEC isolates exhibit biofilm-forming ability, and strong biofilm producers are often associated with higher levels of antimicrobial resistance and persistence of infection*.* The coexistence of multiple virulence factors further enhances the pathogenic potential of these strains, particularly in complicated and catheter-associated UTIs. Existing literature highlights the importance of continuous surveillance, rational antibiotic use, and effective infection control measures in managing UPEC-associated UTIs, particularly in tertiary care settings. Understanding the interplay between antimicrobial resistance, virulence determinants, and biofilm formation is essential for improving clinical outcomes and guiding therapeutic strategies in the management of UTIs.

## Introduction and background

Worldwide, urinary tract infections (UTIs) are among the most prevalent bacterial infections, impacting people across all age groups and genders [1,2]. Each year, around 150 million cases are documented globally, contributing significantly to morbidity, healthcare utilization, and economic burden [3,4]. The inappropriate and excessive use of antibiotics has accelerated the occurrence of antimicrobial-resistant uropathogens, making treatment more difficult and increasing the likelihood of recurrent and persistent infections [5,6]. In India, UTIs represent a significant public health issue and are frequently encountered in clinical practice, particularly among women, elderly individuals, diabetic patients, and individuals requiring long-term urinary catheterization [7,8]. Recent studies from India highlight a concerning rise in antimicrobial resistance among uropathogens, challenging both empirical treatment approaches and infection prevention practices within hospitals [9,10]. Among the various causative agents, *Escherichia coli* is the predominant pathogen, responsible for 70-95% of community-acquired and 40-60% of nosocomial UTIs globally [3,11]. Similar trends are observed in Indian healthcare settings [12]. Although *E. coli* is a naturally occurring commensal of the intestinal microbiota, certain strains have evolved specialized virulence factors enabling them to cause extraintestinal infections, particularly in the urinary tract [13,14]. Uropathogenic *Escherichia coli* (UPEC), a subgroup of extraintestinal pathogenic *E. coli *(ExPEC), possesses multiple virulence determinants, including adhesins, toxins, iron acquisition systems, and serum resistance factors, that facilitate colonization, invasion, immune evasion, and tissue damage [15,16]. UPEC causes a spectrum of clinical infections, including uncomplicated cystitis, pyelonephritis, and urosepsis [3,17]. While community-acquired infections are typically uncomplicated, hospital-associated UTIs are often complicated and occur among individuals with predisposing factors such as prolonged hospitalization, catheterization, structural abnormalities, and immunosuppression [3,11]. Healthcare settings play an important role in the emergence of multidrug-resistant (MDR) UPEC strains due to factors such as extensive antibiotic use and invasive procedures, including strains producing extended-spectrum beta-lactamases (ESBLs) and carbapenemases [18,19].

Comprehensive characterization of UPEC is key to understanding its role in disease severity and management. Analysis of antimicrobial resistance patterns aids in guiding effective therapy and antimicrobial stewardship [20,21]. Evaluation of pathogenic determinants and biofilm formation provides important insights into UPEC pathogenesis, persistence, and associated treatment challenges, particularly in catheter-associated infections [3,22]. Understanding the interplay between resistance, virulence, and biofilm formation is essential for enhancing patient outcomes and implementing effective infection control measures in tertiary care hospitals [23].

## Review

Search strategy

A systematic literature search was conducted in accordance with the Preferred Reporting Items for Systematic reviews and Meta-Analyses guidelines across electronic databases, including PubMed, Scopus, Web of Science, and Google Scholar. Relevant keywords and MeSH terms such as “uropathogenic Escherichia coli,” “UPEC,” “urinary tract infection,” “antimicrobial resistance,” “extended-spectrum β-lactamase,” “virulence genes,” and “biofilm formation” were used. Boolean operators (AND, OR) were applied to refine the search. Studies were screened based on predefined inclusion and exclusion criteria, and duplicates were removed.

**Figure 1:**
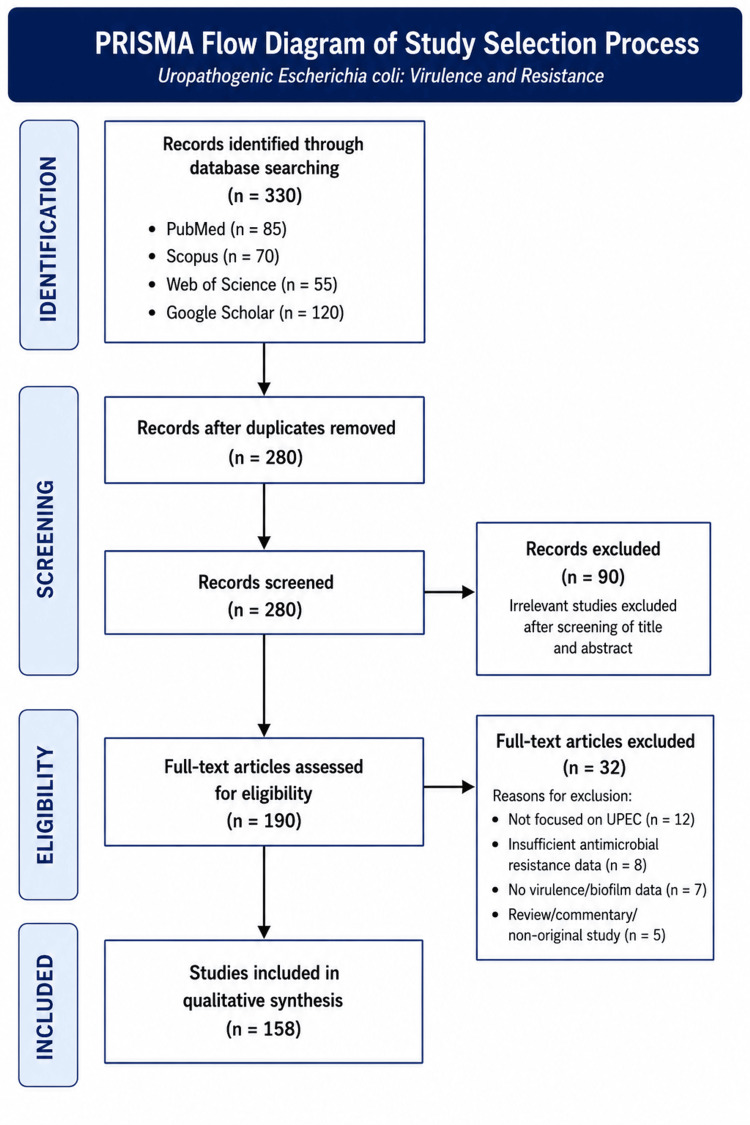
Preferred Reporting Items for Systematic Reviews and Meta-Analyses (PRISMA) flow diagram.

Articles were screened based on their titles and abstracts, followed by a full-text review of potentially relevant studies. The initial database search identified 330 records for analysis. After removing duplicate records, 280 articles remained for screening. During title and abstract screening, 90 records were excluded because they were not relevant to the study topic. Subsequently, 190 full-text articles were assessed for eligibility. Of these, 32 articles were excluded due to a lack of focus on UPEC, insufficient antimicrobial resistance data, absence of virulence or biofilm-related information, or because they were review articles, commentaries, or other non-original studies. Finally, 158 studies met the inclusion criteria and were included in the qualitative synthesis. The schematic representation of the literature selection process is presented in Figure 1.

Global and clinical epidemiology of uropathogenic *Escherichia coli*

Globally, UPEC remains the predominant pathogen responsible for UTIs [3]. Recent studies indicate that UPEC accounts for approximately 75-90% of community-acquired UTIs and around 30-50% of hospital-acquired infections, underscoring its major role in both uncomplicated and complicated cases [11]. Numerous epidemiological investigations across diverse geographic regions and healthcare settings consistently confirm the predominance of *E. coli *among uropathogens [24].

Prevalence of uropathogenic *Escherichia coli* among urinary tract infection isolates

Prevalence rates of UPEC among culture-confirmed UTI isolates vary across different patient groups and clinical settings [25]. In community-based infections, UPEC remains the predominant pathogen, particularly among females, owing to its ability to colonize the peri-urethral area and ascend the urinary tract; although less frequent, it is also a significant cause of UTIs in males [26]. Within hospital settings, although other organisms such as *Klebsiella* spp, *Enterococcus* spp, and *Pseudomonas aeruginosa* are commonly encountered, UPEC continues to be the most commonly isolated individual pathogen [27]. Recent studies from tertiary care centers in India report that *E. coli* remains the predominant uropathogen, accounting for approximately 60-75% of urinary isolates, thereby confirming its epidemiological predominance in both community and hospital settings [28].

Distribution of uropathogenic Escherichia coli in various clinical settings

The occurrence of pathogenic *E. coli *of the urinary tract varies based on the route of infection acquisition and clinical setting [29].

Community-Acquired Urinary Tract Infections

UPEC represents the leading etiological agent in community-acquired UTIs, especially prevalent among young and middle-aged women. These infections are typically uncomplicated and are linked to strains with enhanced adhesion and colonization capabilities [30].

Hospital-Acquired Urinary Tract Infections

In healthcare-associated environments, UPEC continues to be a significant pathogen and is increasingly linked to multidrug resistance due to sustained antibiotic exposure and extended hospital stays. Such infections are often complicated and may result in poorer clinical outcomes [31].

Catheter-Associated Urinary Tract Infections

UPEC is among the predominant organisms responsible for device-associated UTIs. Its capacity to form biofilms on long-term urinary catheters promotes persistence, decreases antimicrobial susceptibility, and contributes to recurrent infections, posing a major challenge in tertiary care settings [32].

Risk factors: demographic and clinical perspectives

A variety of demographic and clinical parameters act as risk factors in determining the occurrence and distribution of UPEC infections [33].

Age and Sex

Lower and upper UTIs are more frequently observed in females due to anatomical characteristics such as a shorter urethra, whereas elderly individuals of both genders are at higher risk owing to underlying comorbid conditions and functional abnormalities of the urinary tract [33].

Diabetes Mellitus and Immunosuppression

Individuals with diabetes mellitus or compromised immune status are more prone to UPEC infections, primarily due to reduced host defense mechanisms and conditions that favor bacterial proliferation [29].

Hospitalization and Catheterization

Published evidence identifies indwelling urinary catheterization, along with prolonged hospitalization and intensive care unit stay, as major risk factors for UPEC-associated infections, particularly catheter-associated UTIs and MDR strains [32].

Demographic and clinical risk factors

Published literature identifies several key demographic and clinical factors associated with an increased risk of UPEC infections. Female sex remains a major predisposing factor due to anatomical susceptibility, while age-related factors contribute to increased risk in both pediatric and elderly populations. Comorbid conditions such as diabetes mellitus and immunosuppression further increase susceptibility and are often associated with complicated infections. In healthcare settings, prolonged hospitalization, intensive care unit stay, and indwelling urinary catheterization are well-established risk factors, particularly for MDR UPEC strains. These factors often act synergistically, contributing to both infection risk and adverse clinical outcomes [34].

Epidemiological Trends in Tertiary Care Settings

Recent epidemiological data indicate increasing antimicrobial resistance among UPEC, with higher resistance rates reported in hospital-based isolates compared to community settings, particularly in patients with prior antibiotic exposure and invasive interventions. Studies indicate that tertiary care hospitals report a higher burden of MDR UPEC compared to community settings, largely due to increased antibiotic pressure and invasive procedures, with a substantial proportion of isolates exhibiting ESBL production [35]. Evidence from India as well as other regions indicates a rising prevalence of ESBL-producing and carbapenem-resistant UPEC, particularly among hospitalized patients and those with indwelling urinary catheters [36]. These emerging trends underscore the growing clinical and public health significance of UPEC in tertiary care settings and highlight the necessity for continuous surveillance and effective infection control strategies [37].

Distribution and Epidemiology of Uropathogenic Escherichia coli

Recent global data consistently demonstrate that UPEC remains the leading cause of community-acquired UTIs, contributing to approximately 75-90% of cases, with many contemporary studies reporting prevalence close to 80-85% [35]. In healthcare-associated settings, UPEC is responsible for nearly 40-60% of nosocomial UTIs, particularly in patients presenting with complicated conditions [31]. Catheter-associated UTIs constitute the majority of hospital-acquired UTIs, with *E. coli* frequently identified as the predominant pathogen [38]. The incidence of UPEC infections is notably higher in tertiary care hospitals, where patients are frequently exposed to prior antibiotic therapy, prolonged hospitalization, and multiple comorbid conditions [39]. Demographic variables such as female gender, increasing age, pregnancy, and postmenopausal status are well-established risk factors, contributing to UPEC-associated UTIs [33]. Chronic comorbid conditions are recognized risk factors for recurrent and complicated UPEC infections [40]. Reports from India and other developing regions indicate a concerning increase in MDR UPEC isolates in tertiary care settings, posing significant challenges for empirical treatment and infection control practices [31].

Molecular mechanisms of antimicrobial resistance

Recent literature indicates that, despite rising multidrug resistance among UPEC, nitrofurans (e.g., nitrofurantoin) and fosfomycin continue to retain relatively good activity [41]. Studies from India and other regions report nitrofurantoin susceptibility rates of approximately 70-85%, with resistance ranging from 10% to 30%, although a gradual increase in minimum inhibitory concentrations (“MIC creep”) has been noted [42]. Similarly, fosfomycin demonstrates high efficacy, with susceptibility rates of 90-96% and low resistance rates of around 4-10%, even among ESBL-producing UPEC isolates [43]. These findings support their continued role as important therapeutic options, particularly for uncomplicated UTIs, although emerging resistance trends warrant ongoing surveillance [44]. Plasmid-mediated resistance mechanisms, including *mcr* genes, confer resistance to colistin, further restricting last-resort therapeutic options [45].

Mechanisms of Resistance

Antimicrobial resistance in UPEC is predominantly mediated by the production of ESBLs, with *blaCTX-M* being the most prevalent gene, followed by *blaTEM* and *blaSHV* [46]. Emerging carbapenem resistance, particularly in hospital settings, is largely associated with carbapenemase genes such as *blaNDM*, *blaOXA-48*, and *blaKPC* [47]. Fluoroquinolone resistance is mainly driven by mutations in the quinolone resistance-determining regions of *gyrA* and *parC* [48]. Additional mechanisms, including efflux pump overexpression and porin loss, further contribute to reduced intracellular antibiotic accumulation [49].

Clinical implications

Resistance to antimicrobials in UPEC significantly contributes to treatment failures and recurrent UTIs. Empirical therapy is essential in the initial management of UTIs, but inappropriate use without susceptibility guidance may lead to treatment failure and resistance; moreover, complicated UTIs can result in severe outcomes such as pyelonephritis and urosepsis [50]. Infections caused by MDR and extensively drug-resistant UPEC strains are linked to prolonged hospitalization, increased healthcare expenditure, and higher morbidity, especially in tertiary care settings [51]. The growing prevalence of ESBL-producing and carbapenem-resistant UPEC has markedly reduced the effectiveness of standard antimicrobial regimens, often necessitating the use of last-resort agents such as colistin, tigecycline, and newer β-lactam/β-lactamase inhibitor combinations [52]. Recent global trends indicate emerging resistance even to last-line antibiotics such as colistin, often mediated by *mcr* genes, underscoring the need for strengthened antimicrobial stewardship [53].

Virulence factors of uropathogenic *Escherichia coli*

Adhesins

Adhesins are essential virulence factors that allow UPEC to adhere to uroepithelial surfaces and avoid being flushed out by the flow of urine. Type 1 fimbriae, mainly encoded by the *fimH* gene, promote mannose-sensitive attachment to bladder epithelial cells and are crucial in the development of cystitis as well as the formation of intracellular bacterial communities [54]. P fimbriae, governed by the *pap* gene cluster, enable binding to epithelial cells of the kidney and are closely linked with pyelonephritis and infections of the upper urinary tract [13]. S fimbriae, along with adhesins, assist in adhesion to renal epithelial and vascular endothelial cells and are frequently identified in UPEC strains responsible for complicated and recurrent UTIs [55].

Toxins

Toxins produced by UPEC contribute significantly to host tissue damage and play a central role in the pathogenesis of UTIs [56]. Alpha-hemolysin, encoded by the *hlyA* gene, is a pore-forming toxin that causes host cell lysis, inflammation, and injury to urinary tract tissues [57]. Cytotoxic necrotizing factor-1 alters host cell signaling pathways, promotes bacterial invasion, and enhances persistence within uroepithelial cells [58].

Mechanisms of Iron Acquisition in Uropathogenic Escherichia coli

Iron acquisition mechanisms are crucial for the survival and growth of UPEC in the iron-restricted environment of the urinary tract [59]. Aerobactin, encoded by the *iuc* gene cluster, acts as a high-affinity siderophore and is commonly linked with highly virulent UPEC strains [60]. Enterobactin, along with other siderophore systems, contributes to enhanced bacterial proliferation, persistence, and competitive fitness during infection [61].

Protectins and Invasins

Capsular polysaccharides, encoded by *kps* genes, provide protection to UPEC against phagocytosis and complement-mediated lysis, thereby promoting persistence and systemic dissemination [62]. The serum resistance-associated gene *traT* plays an important role in helping the bacteria evade host immune responses and supports survival in both bloodstream and urinary tract infections [63].

Prevalence and distribution of virulence genes in uropathogenic Escherichia coli

The distribution of virulence genes among UPEC isolates varies widely depending on the site of infection, severity of disease, and host-related factors such as age, immune status, and comorbidities, as well as geographical differences [30]. Comparative analyses across studies indicate that UPEC isolates from complicated, recurrent, and catheter-associated UTIs harbor a higher prevalence and diversity of virulence genes than those from uncomplicated infections. These strains frequently carry multiple virulence determinants, including adhesins, toxins, iron acquisition systems, and protectins, which collectively enhance pathogenicity and persistence [64]. Furthermore, several studies have demonstrated a strong association between specific virulence gene profiles and increased disease severity, recurrence, antimicrobial resistance, biofilm formation, and adverse clinical outcomes, highlighting their potential role as prognostic markers and therapeutic targets [65].

Biofilm-producing capabilities of uropathogenic *Escherichia coli*

Biofilm formation in UPEC is a highly organized process in which bacterial cells attach to living or non-living surfaces and become enclosed within a self-produced extracellular polymeric substance (EPS) matrix [66]. The process of biofilm development proceeds through distinct stages, namely, initial attachment, irreversible adhesion, maturation, and dispersion [3]. In the urinary tract, the ability of UPEC to form biofilms on uroepithelial surfaces and indwelling urinary catheters enhances persistence and contributes to chronic and relapsing infections [67]. Biofilm formation serves as a key survival strategy, enabling evasion of host immune responses and reduced susceptibility to antimicrobial agents [68]. The EPS matrix further limits antibiotic penetration and facilitates horizontal gene transfer, promoting antimicrobial resistance [69]. In laboratory settings, biofilm production is commonly assessed using phenotypic methods such as the Congo red agar (CRA) method and the microtiter plate (crystal violet) assay. While the CRA method is a simple qualitative technique for screening biofilm producers based on colony morphology, the microtiter plate assay provides a more sensitive and quantitative measurement of biofilm biomass and is considered the gold standard for in vitro assessment [70]. Recent studies indicate that strong biofilm-forming UPEC isolates are frequently associated with multidrug resistance and are more commonly implicated in recurrent, catheter-associated, and complicated UTIs, contributing to increased morbidity and healthcare burden [71].

Molecular characterization of uropathogenic Escherichia coli

Molecular characterization plays a crucial role in identifying antimicrobial resistance determinants, virulence-associated genes, and biofilm-related factors in UPEC isolates from tertiary care settings. Polymerase chain reaction (PCR) is widely employed for the detection and characterization of these genetic markers [72]. ESBL genes such as *blaTEM*, *blaSHV*, and *blaCTX-M* are commonly detected in UPEC isolates and are responsible for resistance to β-lactam antibiotics, particularly third-generation cephalosporins [73]. Plasmid-mediated quinolone resistance genes, including *qnrA*, *qnrB*, and *qnrS*, contribute to reduced susceptibility to fluoroquinolones and are frequently associated with MDR UPEC strains. These genes are commonly detected using molecular methods such as PCR and are increasingly utilized in antimicrobial resistance surveillance programs due to their role in horizontal gene transfer and early detection of emerging resistance. Comparative studies indicate that while the prevalence of *qnr* genes is generally lower than that of β-lactamase genes such as *blaCTX-M*, they often coexist with other resistance determinants, thereby contributing to the MDR phenotype and facilitating the spread of resistance across bacterial populations [74]. Aminoglycoside-modifying enzyme genes such as *aac(6′)-Ib* and *ant(3″)-II* play a significant role in conferring resistance to aminoglycoside antibiotics [75]. Adhesion-related virulence genes such as *fimH* and *fimA* encode components of type 1 fimbriae, facilitating bacterial attachment to uroepithelial cells and promoting colonization [76]. P fimbrial genes, including *papEF*, *papC*, and *papAH*, are involved in pyelonephritis-associated adhesion and contribute to upper UTIs [77]. Other adhesin genes, such as *sfaS*, *bmaE*, and *foc/G*, are associated with S fimbriae and related structures that enhance bacterial adherence and virulence in complicated infections [78].

Epidemiological findings from tertiary care hospitals

Investigations conducted in tertiary care hospitals consistently demonstrate a high burden of antimicrobial-resistant UPEC among hospitalized patients, particularly in complicated cases and catheter-associated UTIs, where UPEC accounts for approximately 60-75% of isolates [79]. A large proportion of these isolates exhibit multidrug resistance, reported in nearly 40-70% of cases, often with the coexistence of multiple virulence determinants that enhance pathogenicity and persistence [80]. ESBL-producing UPEC strains are widely reported in tertiary healthcare centers, with prevalence rates ranging from 40% to 60%, contributing significantly to resistance against third-generation cephalosporins [81]. Although less prevalent than ESBL producers, carbapenem-resistant UPEC isolates have been increasingly identified, with reported rates of approximately 5-15%, particularly in intensive care units and high-dependency settings [82]. The use of indwelling urinary catheters is a major risk factor, with studies indicating that 50-80% of catheter-associated isolates demonstrate biofilm-forming ability and increased resistance [83]. Prolonged hospitalization, especially beyond 7-10 days, further elevates the risk of acquiring resistant UPEC due to sustained exposure to hospital flora and antibiotic pressure [84]. Additionally, the irrational or excessive use of broad-spectrum antibiotics has been strongly linked to the emergence and spread of MDR UPEC strains in tertiary care settings [85]. Surveillance studies from India indicate higher resistance rates to fluoroquinolones and third-generation cephalosporins compared to many Western countries, with reported resistance rates of approximately 60-80% for fluoroquinolones and 50-70% for third-generation cephalosporins, whereas lower rates of around 20-40% are commonly reported in several Western settings [86]. These differences largely reflect variations in antibiotic usage patterns, availability of over-the-counter antibiotics, and antimicrobial stewardship practices. Globally, an increasing trend of community-acquired infections caused by hospital-associated resistant UPEC clones has been observed, with studies suggesting that 10-30% of community isolates may now harbor resistance traits traditionally associated with hospital strains, emphasizing the need for effective infection control measures and antimicrobial stewardship programs [87].

Clinical and public health implications

The rising prevalence of antimicrobial-resistant UPEC has posed major challenges in the effective treatment of UTIs, especially in tertiary care hospital settings [87]. Increasing resistance to commonly used first-line agents such as fluoroquinolones, cotrimoxazole, and third-generation cephalosporins has been associated with higher rates of empirical therapy failure. Critically, this trend reflects not only the widespread and often inappropriate use of antibiotics but also the limited availability of updated local antibiograms to guide empirical therapy. In addition, most available data are derived from hospital-based studies, which may overestimate resistance rates compared to community settings, thereby limiting generalizability. These findings underscore the need for region-specific surveillance, rational antibiotic prescribing, and periodic revision of treatment guidelines to improve clinical outcomes and reduce the burden of resistance [88]. The emergence of ESBL and carbapenemase-producing UPEC strains has further restricted available treatment options, often requiring the use of last-resort or combination antimicrobial therapies [89]. These resistant infections are associated with prolonged hospital stays, increased healthcare expenditure, and higher morbidity among affected patients [89]. Implementation of targeted antimicrobial stewardship programs is essential to promote rational antibiotic use, minimize selective pressure, and control the spread of MDR organisms [90]. Adoption of evidence-based infection control practices, including proper hand hygiene, aseptic catheter insertion, and timely removal of indwelling catheters, is critical in preventing hospital-acquired and catheter-associated UTIs [91]. Ongoing surveillance of local antimicrobial resistance patterns is vital for guiding empirical treatment decisions and updating hospital antibiotic policies [92]. Advanced diagnostic approaches, particularly molecular techniques for detecting resistance and virulence genes, enhance clinical decision-making by enabling early and precise therapy, thereby improving patient outcomes [93]. However, a key contradictory aspect is that despite their high sensitivity and rapid turnaround, molecular methods may not always correlate with phenotypic resistance, and their limited availability, higher cost, and inability to detect novel or uncharacterized resistance mechanisms can restrict their routine clinical utility.

## Conclusions

UPEC is the leading cause of UTIs in both community and hospital settings, particularly in tertiary care centers. The increasing prevalence of MDR strains, especially ESBL and emerging carbapenemase producers, along with key virulence factors such as adhesins, toxins, iron acquisition systems, and biofilm formation, contributes to increased disease severity, recurrence, and treatment failure. These trends highlight the need for continuous surveillance, routine antimicrobial susceptibility testing, and molecular characterization to guide appropriate therapy. Strengthening antimicrobial stewardship through antibiogram-guided therapy, culture-based antibiotic use, de-escalation strategies, regular audits, and adherence to institutional policies is essential. In addition, strict infection control measures, including hand hygiene and catheter care bundles, along with periodic updates of treatment guidelines, are crucial to reduce infection burden and preserve antimicrobial efficacy.
